# Learned stressor resistance requires extracellular signal-regulated kinase in the prefrontal cortex

**DOI:** 10.3389/fnbeh.2014.00348

**Published:** 2014-10-02

**Authors:** John P. Christianson, Johanna G. Flyer-Adams, Robert C. Drugan, Jose Amat, Rachel A. Daut, Allison R. Foilb, Linda R. Watkins, Steven F. Maier

**Affiliations:** ^1^Center for Neuroscience, Department of Psychology and Neuroscience, University of ColoradoBoulder, CO, USA; ^2^Department of Psychology, Boston CollegeChestnut Hill, MA, USA; ^3^Department of Psychology, University of New HampshireDurham, NH, USA

**Keywords:** rat, prefrontal cortex, ERK, learned helplessness, resilience, plasticity

## Abstract

Behaviorally controllable stressors confer protection from the neurochemical and behavioral consequences of future uncontrollable stressors, a phenomenon termed “behavioral immunization”. Recent data implicate protein synthesis within the ventromedial prefrontal cortex (mPFC) as critical to behavioral immunization. Adult, male Sprague-Dawley rats were exposed to a series of controllable tailshocks and 1 week later to uncontrollable tailshocks, followed 24 h later by social exploration and shuttlebox escape tests. To test the involvement of N-methyl-D-aspartate receptors (NMDARs) and the extracellular signal-regulated kinase (ERK) cascade in behavioral immunization, either D-AP5 or the MEK inhibitor U0126 was injected to the prelimbic (PL) or infralimbic (IL) mPFC prior to controllable stress exposure. Phosphorylated ERK and P70S6K, regulators of transcription and translation, were quantified by Western blot or immunohistochemistry after controllable or uncontrollable tailshocks. Prior controllable stress prevented the social exploration and shuttlebox performance deficits caused by the later uncontrollable stressor, and this effect was blocked by injections of D-AP5 into mPFC. A significant increase in phosphorylated ERK1 and ERK2, but not P70S6K, occurred within the PL and IL in rats exposed to controllable stress, but not to uncontrollable stress. However, U0126 only prevented behavioral immunization when injected to the PL. We provide evidence that NMDAR and ERK dependent signaling within the PL region is required for behavioral immunization, a learned form of stressor resistance.

## Introduction

Stressor exposure is a trigger for a number of psychopathologies, yet some individuals appear to either avoid or recuperate from stressor-induced changes more readily than do others (Southwick et al., 2005; Southwick and Charney, 2012). A considerable effort has been made to identify the ways that environmental, genetic and behavioral factors favor resilience to stressors (Feder et al., 2009; Dudley et al., 2011; Cabib et al., 2012; Franklin et al., 2012; Russo et al., 2012; Scharf and Schmidt, 2012). The degree to which an individual can manipulate the onset, offset, or intensity of a stressor gives rise to the perception of behavioral control, and behavioral control is one factor that mitigates the impact of stressors (Maier and Watkins, 2010; Hammack et al., 2012; Christianson and Greenwood, 2014).

To experimentally study behavioral control we employ a design in which one subject can terminate each of a series of stressors by performing a wheel-turn escape response (Escapable Stress, ES) and one subject is yoked to the ES subject and receives identical stressor exposure but cannot control offset (Inescapable Stress, IS). Behavioral control prevents the immediate consequences of the stressor on fear (Maier, 1990; Baratta et al., 2007), anxiety (Short and Maier, 1993; Christianson et al., 2008), and learning (Jackson et al., 1980). Behavioral control also causes a long-lasting, learned form of stressor resistance termed “behavioral immunization” in which one exposure to ES prevents the consequences of IS that may occur *several days later* (Williams and Maier, 1977; Amat et al., 2006, 2010; Christianson et al., 2008).

Like the acute protective effects of behavioral control (Maier and Watkins, 2010; Hammack et al., 2012; Christianson and Greenwood, 2014), behavioral immunization involves the medial prefrontal cortex (mPFC). Inactivation of the mPFC by muscimol or inhibition of protein synthesis by anisomycin prevented the acquisition of behavioral immunization (Amat et al., 2006). Neurons of the prelimbic (PL), but not infralimbic (IL) mPFC, with projections to the dorsal raphe nucleus (DRN) were activated by both the initial ES and by the subsequent IS (Baratta et al., 2009), and PL layer V pyramidal neurons were more excitable following ES (Varela et al., 2012). These observations suggest that the experience of control causes changes in the mPFC that underlie the long-lasting effects of ES in the behavioral immunization phenomenon.

The present study focused on acquisition of behavioral immunization. The initial encoding of memory clearly involves the N-methyl-D-aspartate receptor (NMDAR; Bliss and Collingridge, 1993; Malenka and Nicoll, 1993; see Morris, 2013 for a recent review). NMDAR activation causes a transient increase in intracellular Ca^2+^ that triggers post-synaptic molecular cascades that further support memory consolidation, leading to new gene transcription and translation (Richter and Klann, 2009). We hypothesized that behavioral immunization depends upon NMDAR activation during ES and the transcription and translation cascades that follow NMDAR activation, namely the extracellular signal-regulated kinases 1 and 2 (ERK1, ERK2) and ribosomal protein kinase S6 (P70S6K), molecular substrates of neuroplasticity (Thomas and Huganir, 2004; Costa-Mattioli et al., 2009).

## Materials and methods

### Behavioral procedures

#### Subjects

Male Sprague-Dawley adult rats (60–70 days old and weighing 275–350 g at the time of testing) served as experimental subjects and juvenile rats (28–32 days old and weighing 90–100 g at the time of testing) were used as stimuli in social interaction tests (Harlan Laboratories, Indianapolis, IN). All rats were allowed a minimum of 7 days to acclimate to the vivarium after arrival and housed in pairs with free access to food and water on a 12 h light/dark cycle. Behavioral procedures were conducted within the first 4 h of the light phase. All procedures were conducted in accordance with the Public Health Service’s (2011) *Guide for the Care and Use of Laboratory Animals* and were approved by the University of Colorado Institutional Animal Care and Use Committee.

#### Stress induction procedures

Rats received ES, inescapable stress (IS) or remained in the home cage (HC). As described previously (Christianson et al., 2010), 25 or 100 electric tail-shocks were administered through copper electrodes, augmented with electrolyte paste, by a Precision Regulated Animal Shocker (Coulbourn Instruments, Whitehall, PA). Rats were restrained by taping the tail to a rod extending from the rear of a 14 × 11 × 17 cm (length by width by height) acrylic box with a wheel 7 cm wide and 9.5 cm in diameter located on the wall opposite the tail. Tailshocks were presented on a variable interval-60 s schedule. For rats that received ES, turning the wheel at the front of the chamber terminated each tail-shock after one-quarter turn of the wheel. If the response was performed within 5 s of shock onset the response requirement doubled for the next trial. This pattern proceeded until a maximum of 4 full wheel-turns was reached. If the response was made after 5 s but before 20 s then the requirement was reduced by half; if no response was made by 30 s the shock terminated and the requirement was reset to one-quarter turn. This procedure is used to insure that the subjects learn an operant response. In order to maintain escape behavior the shock intensity was 1.0 mA for the first 33 trials, 1.3 mA for the following 33 trials and 1.6 mA for the remaining 34 trials. In behavioral immunization experiments, 100 trials of IS (5 s, 1.6 mA) were administered 7 days after ES in restraint tubes as described (Amat et al., 2006).

#### Social exploration test

As reported in Christianson et al. (2010), each experimental subject was placed into a plastic tub cage with shaved wood bedding and a wire lid 60 min before the test. To begin the test a 28 (±2) day-old juvenile was introduced to the cage for 3 min and exploratory behaviors (sniffing, pinning, and allogrooming) initiated by the adult were timed by an observer blind to treatment. Juveniles were used for multiple tests but were never used more than once for the same adult rat.

#### Shuttlebox escape test

As described in Amat et al. (2006), but without fear testing, rats were placed into a two-way shuttle box (Coulbourn Instruments). After 5 min, 5 one-way escape trials were presented in which a single cross of the shuttle box terminated a 0.7 mA scrambled footshock. These were followed by 25 two-way trials in which the rat had to cross the box two times to terminate shock. Typically, it is these double-crossing trials that reveal the impact of prior IS. Trials were presented on a variable 60 s intervals. Crossings were determined by infrared photo switches and escape latencies were computed by the software program Graphic State 3.0 (Colbourn Instruments).

### Western blot

Rats were briefly anesthetized with isoflurane, decapitated and brains were flash frozen in isopentane (−40°C) within 30 s of isoflurane exposure. Bilateral micropunches (1 mm^3^) were collected while the brain was mounted on a freezing cryostat from the regions of interest (Figure 2) and stored at −80°C. A 60 μm section containing the punched region was stained with cresyl violet to verify the punch location within the region of interest. Protein was extracted in lysis buffer (FN0071, Life Technologies, Grand Island, NY) containing phosphatase and protease inhibitors (cOmplete and phosSTOP, Roche, Indianapolis, IN). Total protein was quantified with bicinchoninic acid assay and 10 μg/sample was separated via SDS-PAGE chromatography on a 4–12% Bis-Tris gel (Life Technologies) and transferred to nitrocellulose (iBlot, Bio-Rad, Hercules, CA). Blots were incubated with mouse anti-phospho-p44/42 (1:1000, Cell Signaling #9106, Danvers, MA) and rabbit anti-total-p44/42 (1:10,000, Cell Signaling #4695) stripped (NewBlot, Li-Cor, Lincoln, NE) and re-probed with rabbit anti-phospho-P70S6K (Thr412, 1:1000, Millipore #04–393, Billerica, MA) and mouse anti GAPDH (1:100,000, Abcam #AB8245, Cambridge, MA) antibodies at 4°C overnight. Infrared (680/800 nm) secondary antibodies (1:200, 2 h, room temperature, Li-Cor) were used and emission was quantified using a Li-Cor scanner and software. Tissue punches were made within 7 days of tissue collection and SDS-PAGE/Western blotting occurred within 1 month of the collection to limit degradation of phosphorylated proteins.

**Figure 1:**
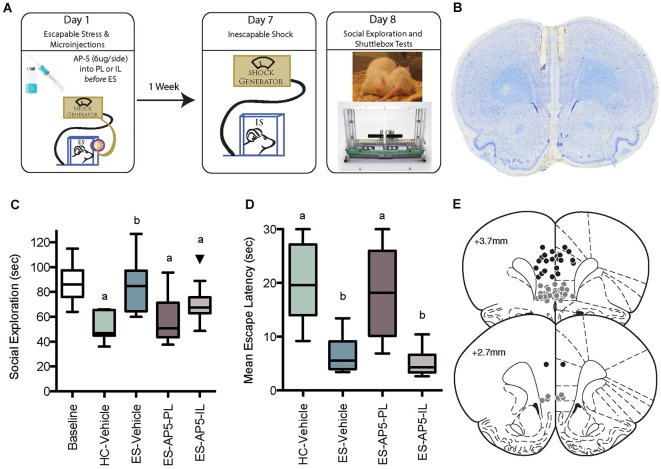
**(A)** Illustration of behavioral immunization procedure. Rats in the HC-Vehicle group were returned to the vivarium after injection on Day 1. All rats received inescapable shock (IS) on Day 7 and behavioral testing on Day 8. **(B)** Photomicrograph depicting a cresyl violet stained section depicting typical cannula tracks terminating in the PL. **(C)** Box and whisker plots depict the minimum, maximum, median and interquartile range of time spent in social exploration (see Statistics Section). One outlier is depicted with a black triangle. Baseline social exploration values were recorded after surgery but prior to any stressor exposure and are shown as a reference. IS significantly reduced social exploration (HC-Vehicle group) whereas rats that received escapable stress (ES-Vehicle) 1-week prior to IS behaved equally to baseline. Rats that received AP5 injections to IL or PL prior to ES behaved akin to the IS treated rats. Plots with different letters were found to have significantly different means, *p*s < 0.05. **(D)** Box and whisker plots of the mean escape latencies computed from 25 two-way escape trails in the shuttlebox test. Failure to escape on a single trial resulted in a 30 s latency, thus the HC-Vehicle and ES-AP5-PL groups did not reliably learn to escape in the task whereas rats with prior ES-Vehicle or ES-AP5-IL demonstrate behavioral immunization with short escape latencies. Groups marked with different letters were significantly different, *p*s < 0.05. **(E)** Cannula tip locations in rats that received prelimbic (PL) microinjections (Black Circles) or infralimbic (IL) injections (Gray Circles). Coronal sections drawn from the Paxinos and Watson (1998, with permission) atlas.

**Figure 2:**
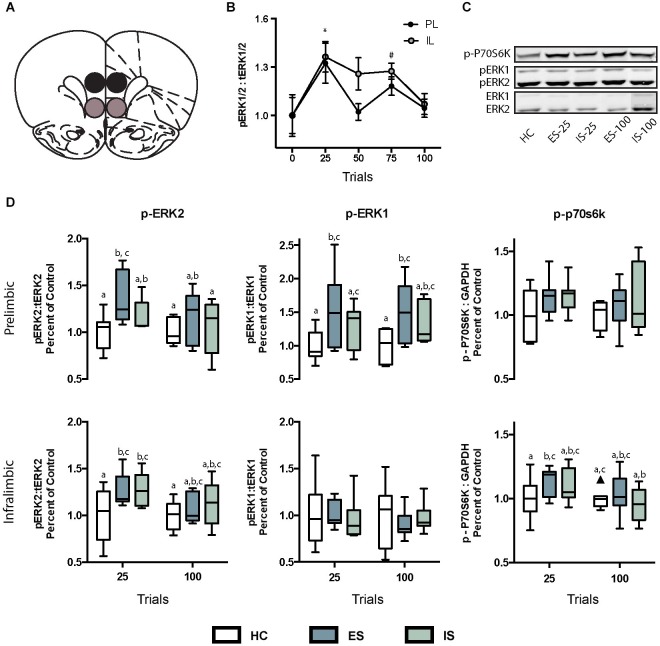
**(A)** Digital template reproduced from the atlas of Paxinos and Watson (1998, permission pending) depicting the location of micropunches from the PL (black circles) or IL (gray circles) regions of interest. **(B)** Effect of ES on ratio of phosphorylated extracellular signal-regulated kinases 1 and 2 (pERK1/2) to total (tERK1/2). * In both IL and PL samples, pERK1/2 was significantly greater than baseline at 25 trials, and ^#^ in the IL pERK1/2 was greater than baseline at 75 trials.** (C)** Infrared images representing targeted bands from the PL cortex. **(D)** Box and whisker plots depicting ratios of specific ERK isoforms pERK2:tERK2 and pERK1:tERK1 and p-p70s6k:GAPDH all normalized to home cage (HC) controls following 25 or 100 trials of ES or inescapable stress (IS). Boxes marked with different letters differed significantly from each other (*p*s < 0.05). One outlier is depicted with a black triangle.

### Immunohistochemistry

**Figure 3:**
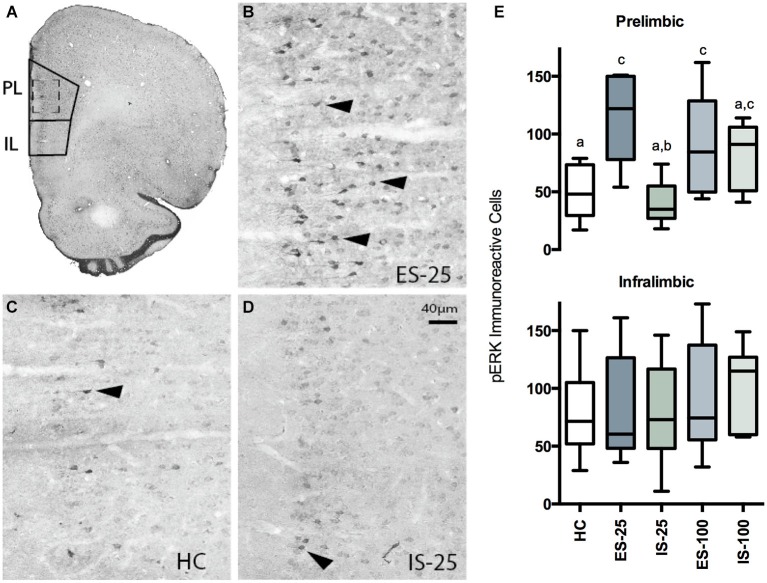
**(A)** Photomicrograph of coronal hemisphere section corresponding to Bregma +2.7 mm. The regions counted for PL and IL cortices are traced in solid lines. **(B–D)** Representative examples from ES-25 **(B)** HC **(C)**, and IS-25 **(D)** rats corresponding to dashed-line box in **(A)**. Phosphorylated extracellular regulated kinase 1/2 (pERK) expression was most abundant within the superficial layers. Characteristic pERK cells are marked with solid arrowheads. **(E)** Box and whisker plots depicting the total number of pERK cells counted from either the PL after HC, ES-25, IS-25, ES-100 or IS-100 treatments. In the PL, ES-25, ES-100 and IS-100 differed from the HC Group while ES-25 differed significantly from the IS-25 group (*p*s < 0.05) indicating a selective effect of stressor controllability.

Rats were deeply anesthetized with sodium pentobarbital (60 mg/kg) and perfused with 100 mL of ice-cold 0.9% saline followed by ~250 mL of 4% paraformaldehyde in 0.1 M phosphate buffer (pH 7.4). Brains were postfixed overnight then transferred to 30% sucrose and stored at 4°C until sectioning. 30 μm sections were obtained in a −20°C cryostat and stored at 4°C in cryoprotectant until staining. Phosphorylated ERK1/2 immunoreactivity was detected by incubating sections with mouse anti-p44/42 MAPK (1:200, 48 h, 4°C, Santa Cruz Biotechnology #SC-7383, Dallas, TX) and biotinylated goat anti-mouse secondary antibody (1:200, 90 min, 4°C, Vector Labs, #BA-9200, Burlingame, CA). Phosphorylated ERK1/2 was visualized with the avidin-biotin horseradish peroxidase method (90 min, 4°C, Vectastain Elite ABC Kit, Vector Labs) and nickel-enhanced 3,3′-diaminobenzidine (Sigma-Aldrich) as chromogen for 10 min at room temperature. One left and one right hemisphere section corresponding to Bregma +2.7 mm were counted for each subject. The rostrocaudal level was determined by comparing an adjacent section stained with cresyl violet to the stereotaxic atlas (Paxinos and Watson, 1998). One observer, blind to group membership, quantified phosphorylated ERK-stained neurons that included a clearly stained cell body and least one dendritic process as shown in Figure 3.

### Intracerebral cannula placement and microinjection

Under inhaled isoflurane (2–5% v/v in O_2_) anesthesia rats were placed in a stereotaxic frame and the skull was leveled by adjusting the incisor so that Bregma and Lambda were in the same horizontal plane. Cannulae (26 g, Plastics One, Roanoke, VA) were inserted bilaterally into the PL or IL (from Bregma: AP: +2.7 mm, ML: +/−0.5 mm, DV: −3.5 mm for PL or −4.5 mm for IL, from skull surface; based on the atlas of Paxinos and Watson, 1998) and fixed in place with acrylic cement and stainless steel screws. Rats were allowed between 7–10 days recovery prior to stress administration. D-AP5 (AP5, Sigma-Aldrich, St. Louis, MO) was dissolved in sterile 0.9% saline at 6 μg/μL as in Bast et al. (2005). U0126 (Tocris, Minneapolis, MN) was dissolved in DMSO and diluted to a final concentration of 1 μg/μL in 0.9% NaCl/25% DMSO as in Schafe et al. (2000). All injections were made with a 25 uL Hamilton syringe in a micromanipulator (Kopf Model 5000) connected to a 33 g injector by PE-50 tubing. Injectors protruded 1 mm past the cannula end and 0.5 μL was infused per side at a rate of and 1 μL/min with an additional minute allowed for diffusion. Rats in the vehicle control groups received equal volume injections of the appropriate diluent: saline for the AP5 experiment or saline with 25% DMSO for the U0126 experiment. Rats were gently restrained in a cloth towel during injections. At the conclusion of the experiment, rats were overdosed with sodium pentobarbital (60 mg/kg) and brains were dissected, sectioned and stained with cresyl violet to verify the microinjector tip location. Rats that received drug injections that fell outside of the IL or PL were excluded from all analyses.

### Experimental designs

The general approach to the behavioral immunization paradigm is to expose the subject to ES on the 1st day, IS on the 7th day and a behavioral test on the 8th day (Figure 1). Importantly, we have repeatedly found that neither restraint, or IS itself given on the first day alter the consequences of subsequent IS (Williams and Maier, 1977; Amat et al., 2006; Christianson et al., 2008). Thus, for simplicity, the experimental control group for ES exposure received the microinjections and handling but were returned to the HC without stressor exposure. With our procedures, IS exposure results in a number of behavioral changes that last a minimum of 24 h. Here we chose to use the juvenile social exploration test and the two-way shuttlebox escape test because IS reliably reduces social exploration (Short and Maier, 1993; Christianson et al., 2008, 2010) and interferes with shuttle escape performance (Maier, 1990; Amat et al., 2005), and these IS effects are mediated by stressor induced sensitization of the serotonergic DRN (Christianson and Greenwood, 2014). Importantly, the effects of IS on DRN sensitization, social exploration and shuttle escape are sensitive to the controllability of prior stress in the behavioral immunization paradigm (Amat et al., 2006; Christianson et al., 2008).

To determine whether NMDARs are necessary for behavioral immunization by ES to the effects of later IS on social exploration and shuttlebox escape, and whether subregions of the mPFC differentially contribute to the effect, rats were implanted with cannulae for injection to either the PL or IL. After recovery, all rats were given a baseline social exploration test and then assigned to treatment groups so that all groups had equal basal social exploration. The distribution of baseline social exploration scores are comparable to what we have published previously and are shown in Figure 1 to provide the reader a reference to evaluate the effects of later stress treatments. We have demonstrated that without stressor exposure (i.e., HC treatment) social exploration levels are stable over 7 days (Christianson et al., 2013). The experimental groups were as follows: HC-Vehicle, ES-Vehicle, ES-AP5-PL, or ES-AP5-IL. The ES-vehicle group included rats with either IL or PL placements. These groups were pooled because in numerous reports, intra IL/PL injections of saline have no effect on the sequella of ES (Amat et al., 2005, 2006; Christianson et al., 2009; Rozeske et al., 2009). Furthermore, pilot data indicated that in unstressed animals, intra-PFC AP5 without stress had no effect on social exploration 8 days later and so this group was not included. On the day after baseline social exploration testing, rats received either AP5 or Vehicle injections 10–15 min prior to ES or return to the HC. Rats in the ES group then received 100 trials of ES and then returned to the vivarium. 7 days later, all rats received 100 trials of IS and on the next day received a social exploration test followed shortly after by two-way shuttlebox escape tests.

Preventing behavioral immunization by intra-PL or IL AP5 suggested that ES causes NMDAR dependent signaling within these regions. NMDAR activation causes transient increases in intracellular Ca^2+^, an important trigger for new protein synthesis cascades. We sought to characterize phosphorylation in the PL and IL of two known regulators of transcription and translation, ERK1/2 and P70S6K. Rats exposed to ES quickly master the wheel-turn escape response, performing maximally within 15–20 trials and it is at about this time in the test when inhibition of the DRN is first detectable, and dependent upon the mPFC (Amat et al., 2005). Thus, the molecular cascades underlying ES are likely engaged relatively early in the ES session. In a pilot study rats were exposed to either no stress or 25, 50, 75, or 100 trials of ES we found that the ratio of phosphorylated ERK (pERK) to total ERK (tERK) increased at the onset of ES, i.e., after 25 trials and appeared to return to baseline (Figure 2B). Thus, rats were assigned to one of six groups in a two-factor, Stress (HC, ES or IS) by Trial (25 or 100) design. Rats were exposed to ES or IS and then anesthetized and decapitated immediately after the 25th or 100th tailshock; rats in the HC groups were removed from the HC and anesthetized without stress exposure. Brains were dissected, flash frozen and processed for Western blot analysis of phosphorylated to total protein ratios of both isoforms (pERK1:tERK1, pERK2:tERK2), phosphorylated P70S6K as compared to loading control GAPDH (p-P70S6K:GAPDH). Tissue micropunches were collected from the PL or IL and three additional regions: anterior cingulate cortex (ACC), dorsomedial striatum (DMS) and dorsolateral striatum (DLS). The ACC was included to determine if stressor controllability activated ERK signaling there, as it could be damaged by the cannula track and diffusion of drugs up the cannula track and into the ACC could partially account for the drug effects found in the PL. The DMS was included because we recently reported that intra-DMS AP5 injections prevented the protective effects of controllable stressor exposure (Amat et al., 2014) and ERK phosphorylation in this region might indicate that DMS, in addition to the mPFC, is a neuroanatomical locus of memory required for behavioral immunization. The DLS was included as a region specificity control.

The Western blot results indicated that ES, relative to IS, selectively increased the ratio of pERK to tERK in the PL. While the Western blot has the advantage of permitting dissociation between ERK1 and ERK2 isoforms, it is difficult to confirm the anatomical specificity of the results as even precise tissue punches may contain small amounts of surrounding tissues. Thus, rats were assigned to HC and either 25 or 100 trials of ES or IS and tissue was extracted and prepared for immunohistochemistry. Representative sections of the mPFC were stained for phosphorylated ERK1/2 as described above.

To test whether the ERK cascade is critical to the behavioral immunization phenomenon, rats were implanted with cannula for microinjection into the PL of the mitogen activated protein kinase kinase (MEK) inhibitor U0126. U0126 is known to prevent phosphorylation of ERK1 and 2 by inhibition of both MEK-1 and MEK-2 kinases (Favata et al., 1998). Although U0126 may prevent phosphorylation of P70S6K (Fukazawa and Uehara, 2000), the Western blot results indicate that ES did not activate this pathway in the PL at the timepoints studied. Rats were then assigned to the following treatments: HC-Vehicle, HC-U0126, ES-Vehicle, ES-U0126, IS-Vehicle, and IS-U0126. A final group was included as an injection site specificity control that received ES and U0126 in the IL. Immediately before ES treatment rats received the appropriate microinjection; rats in the HC and IS groups received injections and then were returned to the vivarium. Rats in the ES groups then received 100 trials of ES, although 9 animals received only 80 trials because of a computer failure. The data from these rats were first compared to the 100 trial group and no differences were found and all rats in the ES group were pooled for analysis. 7 days later, all rats in the ES and IS groups received 100 trials of IS and the next day social exploration was quantified. The number of experimental treatment groups precluded running multiple behavioral tests; social exploration has a number of advantages over the shuttle box (Christianson et al., 2008) and it was used here because it appeared to be sensitive to both PL and IL AP5 manipulations making it more informative than the shuttlebox.

### Statistics

Effects of treatments on group means were assessed with one-way or two-way analysis of variance (ANOVA). Either stress group, drug group or timepoint were considered independent, between-subjects factors in the analyses. When main effects or interactions occurred at *p* < 0.05, Fisher’s PLSD *post hoc* comparisons were made between treatment groups with a threshold of *p* < 0.05. Five descriptive statistics, the minimum, maximum, median, and the 25th and 75th percentiles are depicted in boxplots (Tukey, 1977). If the maximum or minimum data point was greater than or less than 1.5 times the interquartile range plus or minus the 75th or 25th quartile, respectively, then the whisker extends to the quartile plus 1.5 times the interquartile range and the outliers are shown as individual points. Outlying points were excluded from analysis (Tukey, 1977).

## Results

### Intra-PFC AP5 prevented behavioral immunization

Experimental timeline, cannula placements, social exploration and mean shuttle escape latency are depicted in Figure 1; baseline social exploration values are shown as a reference and were not included in the analysis. Exposure to IS alone reduced social exploration from baseline, while prior ES completely prevented this change—this is the behavioral immunization phenomenon. AP5 completely blocked the protective effect of ES when injected to either the PL or IL. Only rats with cannula tips falling within the PL or IL were included for analysis, which resulted in the following groups (n): HC-Vehicle (9), ES-Vehicle (8), ES-AP5-PL (9) and ES-AP5-IL (10). One outlier in the ES-AP5-IL group is shown and was excluded from analysis. These observations are supported by a significant one-way between-subjects ANOVA, *F*_(3, 32)_ = 6.182, *p* = 0.002 and significant *post hoc* pairwise comparisons between ES-Vehicle vs. IS-Vehicle (*p* < 0.001), ES-Vehicle vs. ES-AP5-PL (*p* < 0.01) and ES-Vehicle vs. of ES-AP5-IL (*p* < 0.05). Shortly after the social exploration tests the rats were given shuttlebox tests. Escape latencies for the one-way trials did not differ between groups (data not shown). The mean latency to escape the two-way escape trials is shown in Figure 1. A one-way ANOVA revealed a significant effect of treatment *F*_(3, 32)_ = 15.05, *p* < 0.0001 and *post hoc* tests found significant differences between IS-Vehicle vs. ES-Vehicle, indicating the behavioral immunization effect. With regard to the shuttlebox, only the ES-AP5-PL group differed significantly from ES-Vehicle (*p* < 0.01); the ES-AP5-IL group appeared to be equal to the ES-Vehicle group and differed significantly from both IS-Vehicle and ES-AP5-PL (*p*s < 0.001), indicating that behavioral immunization in the shuttlebox was unaffected by intra-IL AP5 injections.

### Escapable stress induced ERK phosphorylation in the PL and IL

The ratio of the phosphorylated isoform of ERK to the tERK, or phosphorylated P70S6K to GAPDH were computed from the integrated densities and then normalized to HC controls run on the same day, gel and blot as the experimental groups. No treatment was found to have an effect on tERK or GAPDH expression (data not shown). The specific time points for sampling were determined in a pilot study in which exposure to increasing numbers of ES trials (*n* = 5–8 samples per time point) increased the ratio of pERK1/2 to tERK1/2 (Figure 2B). A significant main effect was found for trials, *F*_(4, 52)_ = 4.95, *p* = 0.002 and *post hoc* tests revealed significant increases in pERK1/2 ratio at 25 trials in both the IL and PL samples, and at 75 trials in the IL samples, *p*s < 0.05. To determine whether the increases in pERK observed after ES are the result of stressor controllability tissue samples were collected immediately after either 25 or 100 trials of ES or IS. The sampling distributions in the PL and IL are shown in Figure 2D and means, SEM and *n* for the ACC, DMS and DLS are shown in Table 1. The ratios for each region of interest were analyzed with two-way ANOVAS with Stress (HC, ES, or IS) and Trials (25 or 100) as between-subjects factors. Two groups received no stress (HC groups) but were required to normalize protein expression across multiple days and blots. The resulting groups had *n*s = 6–8. A number of effects of ES appeared in the PL and IL. Significant main effects of stress were found in the PL for pERK2, *F*_(2,34)_ = 3.957, *p* = 0.029 and for pERK1, *F*_(2, 36)_ = 5.644, *p* = 0.007. For pERK2, post hoc comparisons found an increase in ES-25 compared to HC groups and for pERK1 both ES-25 and ES-100 were significantly greater than HC groups, *p*s < 0.05. In IL, a significant main effect of stress was found on pERK2, *F*_(2, 37)_ = 3.798, *p* = 0.032 and a significant main effect of trials was found on p-P70S6K, *F*_(1, 40)_ = 5.304, *p* = 0.027. For IL pERK2 both ES-25 and IS-25 were significantly greater than HC *p*s < 0.05. The main effect of trials in IL p-P70S6K can be attributed to a significant difference between ES-25 and HC and significant differences between IS-100 and both ES-25 and IS-25 groups. In the DMS, main effects of stress were found for pERK1, *F*_(2, 41)_ = 4.852, *p* = 0.013 and p-P70S6K, *F*_(2, 36)_ = 3.976, *p* = 0.028. In the DMS on pERK1 ES-25 and IS-100 were each significantly greater than HC (*p*s < 0.05) and on p-P70S6K both ES-100 and IS-100 were greater than HC (*p*s < 0.05). The ACC and DLS were included as a site specificity controls and no significant effects were found on any measure in these regions. To determine if elevations in phosphorylated protein were related to an ES induced change in tERK expression, tERK1 and tERK2 were normalized to GAPDH and no significant group differences were found (data not shown).

**Table 1:** **Phosphorylated to total protein ratios in additional regions of interest**.

Target	Region	Trials	Treatment		
			*HC*	*ES*	*IS*
pERK2:tERK2	ACC	25	1.0 (0.115), 7	1.131 (0.052), 7	1.092 (0.131), 8
		100	1.0 (0.082), 6	1.041 (0.087), 8	1.176 (0.126), 8
	DMS	25	1.0 (0.091), 7	1.244 (0.138), 8	1.259 (0.170), 8
		100	1.0 (0.054), 8	1.083 (0.092), 8	1.383 (0.164), 8
	DLS	25	1.0 (0.090), 8	1.154 (0.128), 8	1.118 (0.148), 7
		100	1.0 (0.054), 8	1.055 (0.109), 8	1.410 (0.198), 8
pERK1:tERK1	ACC	25	1.0 (0.104), 7	1.173 (0.059), 8	1.118 (0.065), 8
		100	1.0 (0.118), 7	1.197 (0.136), 5	1.317 (0.211), 8
	DMS	25	1.0 (0.027), 7	1.172 (0.074), 8*	1.147 (0.039), 8
		100	1.0 (0.034), 8	1.044 (0.041), 8	1.193 (0.083), 8*
	DLS	25	1.0 (0.101), 8	1.139 (0.114), 7	1.154 (0.068), 8
		100	1.0 (0.047). 8	1.132 (0.060), 7	1.185 (0.075), 8
p-P70S6K:GAPDH	ACC	25	1.0 (0.048), 8	1.151 (0.073), 8	1.069 (0.057), 8
		100	1.0 (0.022), 7	1.019 (0.059), 7	1.022 (0.125), 8
	DMS	25	1.0 (0.045), 6	1.115 (0.065), 8	1.186 (0.077), 8
		100	1.0 (0.049), 5	1.268 (0.088), 8*	1.249 (0.100), 8*
	DLS	25	1.0 (0.059), 6	1.022 (0.060), 7	0.991 (0.048), 8
		100	1.0 (0.063), 8	0.977 (0.044), 7	0.952 (0.030), 8

Mean (SEM) and n for of phosphorylated to total protein ratios in the anterior cingulate cortex (ACC), dorsomedial striatum (DMS) and dorsolateral striatum (DLS) after 25 or 100 trials of home cage (HC), escapable stress (ES) or inescapable stress (IS). * Significantly greater than HC, p < 0.05.

### Immunohistochemical quantification of PL and IL phosphorylated ERK

Box-plots reflecting the total number of pERK immunoreactive cells within the PL and IL regions immediately after HC, ES-25, IS-25, ES-100 or IS-100 treatments (*n*s = 5–8/group) are depicted in Figure 3. A one-way ANOVA revealed a significant main effect in PL, *F*_(4, 29)_ = 5.509, *p* = 0.0032. Significantly more pERK immunoreactive cells were found in the ES-25 and ES-100 groups compared to HC controls and the IS-25 group (*p*s < 0.05). The IS-100 group also differed significantly from the HC group (*p* < 0.05). No main effect was found in the IL, *F*_(4, 29)_ = 0.5759, *p* = 0.682.

### Inhibition of ERK1/2 cascade in the PL prevented behavioral immunization

**Figure 4:**
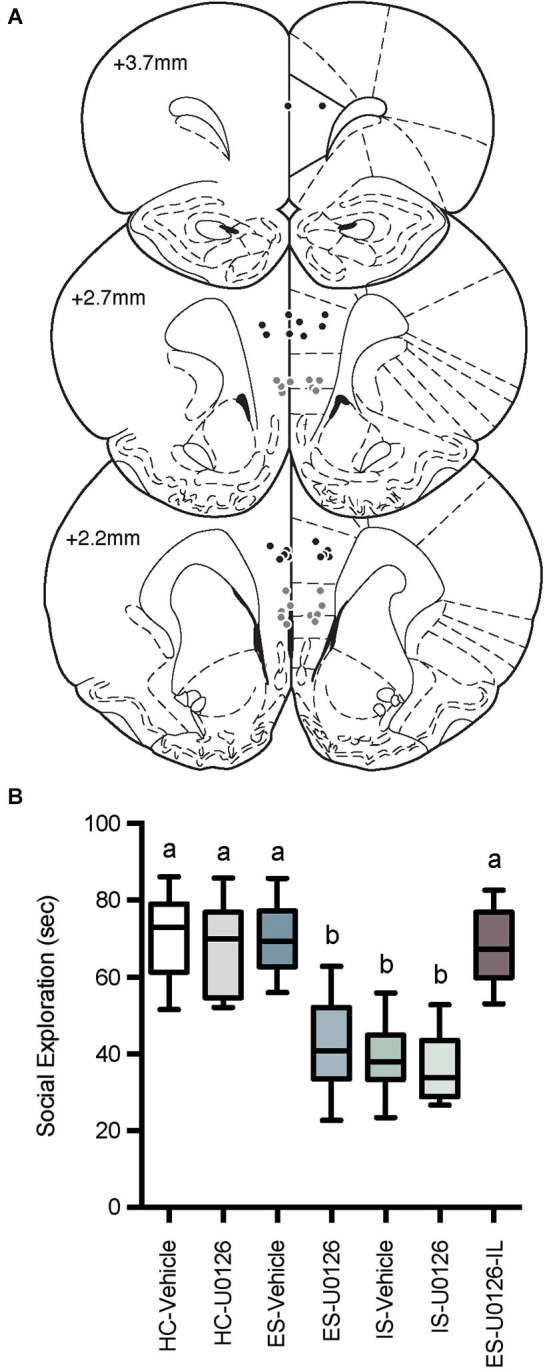
**(A)** Cannula tip locations in rats that received PL microinjections (Black Circles) or IL injections (Gray Circles) of U0126 and ES (drawn from Paxinos and Watson, 1998 with permission). **(B)** Box and whisker plots of time spent in social exploration. All treatment groups received microinjections into the PL except the site specificity control group, which received drug injection in the IL (ES-U0126-IL). The HC groups received microinjections on Day 1 and social exploration on Day 8, the IS groups received microinjections on Day 1, IS on Day 7 and social exploration on Day 8, and the ES groups received microinjections and ES on Day 1, IS on Day 7 and social exploration tests on Day 8. IS significantly reduced social exploration compared to HC groups whereas rats that received ES-Vehicle 1-week prior to IS behaved equally to baseline demonstrating behavioral immunization. Rats that received U0126 injections to the PL (ES-U0126), but not IL (ES-U0126-IL) prior to ES behaved akin to the IS treated rats. Plots with different letters were found to have significantly different means, *p*s < 0.05.

Microinjector locations and social exploration times are depicted in Figure 4. Rats with misplaced cannula were excluded from analysis and groups had the following *n*; HC-Veh, *n* = 10; HC-U0126, *n* = 7; ES-Vehicle, *n* = 9; ES-U0126, *n* = 13; IS-Vehicle, *n* = 10; IS-U0126, *n* = 9; ES-U0126-IL, *n* = 6. A significant main effect of treatment was found with a one-way between subjects ANOVA, *F*_(6, 57)_ = 22.88, *p* < 0.001. *Post hoc* comparisons revealed that social exploration levels in ES-U0126, IS-Vehicle, and IS-U0126 were significantly lower than all other groups (*p*s < 0.05). Thus, in this experiment rats that received prior ES-Vehicle treatment displayed the typical behavioral immunization while rats exposed to IS showed a marked reduction in social exploration. Importantly, intra PL U0126 completely prevented behavioral immunization in the ES Group, while U0126 had no effect in the site specificity control group (ES-U0126-IL).

## Discussion

We provide evidence that the protection from stress that is acquired upon exposure to a controllable stressor, i.e., behavioral immunization, requires synaptic and cellular pathways known to be critical for synaptic plasticity, learning, and memory. Specifically, behavioral immunization requires NMDAR and ERK activation in the mPFC, likely within the PL. These results have implications regarding both the neural machinery that underlies the stressor controllability phenomenon and a number of issues relating to resilience to stress-related psychiatric illness.

Most of the prior reports from our group that have implicated the mPFC in mediating the protective effects of behavioral control did not attempt to distinguish between PL and IL regions. For example, injections of pharmacological agents designed to (a) inhibit mPFC function during the experience of control (muscimol; Amat et al., 2005, 2006; Christianson et al., 2009; Rozeske et al., 2009); (b) activate the mPFC output (picrotoxin; Amat et al., 2008; Christianson et al., 2009; Rozeske et al., 2009); and (c) inhibit mPFC protein synthesis (anisomycin; Amat et al., 2006), were all microinjected at the PL/IL border. However, the PL and IL have distinguishable efferent projections (Sesack et al., 1989; Vertes, 2004; Gabbott et al., 2005) which give rise to a number of functional distinctions (Dalley et al., 2004; Peters et al., 2009; Lasseter et al., 2010). For example, the PL appears to promote the learning and expression of fear, whereas the IL contributes to the inhibition of fear in rodents (Vidal-Gonzalez et al., 2006), and abnormalities in these circuits contribute to PTSD in humans (Milad et al., 2009).

The present experiments were designed, in part, to dissociate the roles of the PL and IL in the stressor controllability paradigm. To this end, each experiment attempted to isolate these regions with site-specific injections, microdissection, or cytoarchitecture. Both intra-PL and intra-IL NMDAR blockade prevented behavioral immunization with regard to social exploration, but only intra-PL infusions affected shuttlebox escape. Thus, it is possible that NMDAR dependent signaling in the IL and PL is critical to behavioral immunization and perhaps IL and PL differentially contribute to stressor effects on different dependent measures. We cannot rule out the possibility that intra-IL drug infusions affected the PL by diffusing up the cannula track. However, evidence from pERK quantificiation and U0126 injection suggests that the PL can be dissociated from the IL with regard to the behavioral immunization phenomenon. ES led to marked increases in pERK:tERK ratios of both isoforms (1/2) in the PL, whereas IS did not. With regard to pERK immunoreactivity, both 25 and 100 trials of ES increased the number of pERK immunoreactive cells in the PL, replicating the effects of ES on protein expression. IS caused a significant increase in pERK2 after 25 trials, and in pERK immunoreactive cells after 100 trials. In the IL, 25 trials of either ES or IS increased pERK2 but no effects were found on pERK immunoreactive cell counts. Overall, the results of the Western blot and immunohistochemistry support the hypotheses that exposure to ES rapidly triggers ERK phosphorylation. Importantly, ES did not elicit clear effects on pERK in the ACC, DMS, or DLS suggesting circuit selectivity.

To determine whether the regionally specific phosphorylation of ERK in the PL was critical to behavioral immunization, the MEK inhibitor U0126 was administered to PL or IL before ES. Consistent with the phosphorylation pattern, U0126 microinjections only interfered with behavioral immunization when injected to the PL; the intra-IL injected group appeared equal to the Vehicle injected ES group. Taken together, this set of studies is consonant with a wealth of data from synaptic plasticity experiments indicating that activity and NMDAR-dependent rises in postsynaptic Ca^2+^ would trigger ERK phosphorylation and subsequent protein translation in support of memory acquisition and consolidation (English and Sweatt, 1996). We found significant increases in both ERK1 and ERK2, but it is difficult to speculate on their relative importance to behavioral immunization for two reasons. First, mounting evidence reveals the complexity and redundancy of ERK regulation and translational control in synaptic plasticity (Selcher et al., 2001; Mazzucchelli et al., 2002). Second, inconsistencies abound in reports indicating both increases and decreases of pERK isoforms after acute or repeated stressors and in a variety of regions of interest (Trentani et al., 2002; Gourley et al., 2008; Iñiguez et al., 2010; Perrotti et al., 2013) and to our knowledge no published work has attempted to determine whether the controllability of the stressor contributed to effects on ERK. Additional studies will be required to determine if ES evoked activity at the NMDAR is causally linked to the ES evoked increase in ERK phosphorylation in the context of behavioral immunization.

What is it about control over stress that engages the PL, leading to resilience? Research in the field of instrumental learning, almost all of which involves appetitive-learning paradigms, suggests that there are two separable systems mediating learned instrumental responses (Balleine and O’Doherty, 2010). One system maintains inflexible, habitual behaviors and involves the sensorimotor cortex and DLS. The other system mediates flexible, goal-directed behaviors that depend upon the relationship between the action and the expected outcome and involves the PL and DMS. It may be that ES activates the PL because ES normally engages learning using the action-outcome contingency (Christianson and Greenwood, 2014), and so the PL/DMS system may be essential for the induction of immunization to later uncontrollable stress. Indeed, when ES subjects were forced to learn using the DLS, by selective inactivation of the DMS during ES, protection from the consequences of tailshocks did not occur (Amat et al., 2014) in press. Increases in ERK signaling occur in the PL after action-outcome learning to avoid an aversive stimulus (Perrotti et al., 2013) and after fear learning (Hugues et al., 2006). These findings lead us to suggest that changes in the PL produced by ES may be a consequence of overlap between fear/stress regulation and action-outcome circuits. Importantly, acute stress *per se* does not necessarily lead to phosphorylation of ERK in the PL (Figure 2 and see Trentani et al., 2002). That is, activation of the PL only occurs because the subject has control over an aversive stimulus and the current studies suggest that NMDAR and ERK dependent mechanisms link these experiential factors in memory. This synaptic connection between behavioral control and emotion regulation could account for the wide range of protective effects of ES including social defeat, forced swimming stress, drug seeking and fear expression (reviewed by Christianson and Greenwood, 2014).

Many psychiatric illnesses triggered by stressor exposure present with impairments in prefrontal cortex function (Drevets et al., 1997; Etkin and Wager, 2007; Milad et al., 2009; Pitman et al., 2012), and rescuing these deficits is a strategic aim of research and development of new therapeutics. Here we provide evidence that a long-lasting behavioral intervention for stress-related pathology depends upon NMDAR and ERK signaling in the prefrontal cortex. This may suggest a molecular and anatomical basis for the effectiveness of cognitive therapies, as they all involve an element of fostering a perception of control. Such therapies could strengthen mPFC top-down inhibitory control over stress responsive limbic and brainstem structures that mediate a range of psychiatric symptoms (DeRubeis et al., 2008).

## Conflict of interest statement

The authors declare that the research was conducted in the absence of any commercial or financial relationships that could be construed as a potential conflict of interest.
